# Mitochondrial networks through the lens of mathematics

**DOI:** 10.1088/1478-3975/acdcdb

**Published:** 2023-07-14

**Authors:** Greyson R Lewis, Wallace F Marshall

**Affiliations:** 1 Biophysics Graduate Program, University of California—San Francisco, San Francisco, CA, United States of America; 2 NSF Center for Cellular Construction, Department of Biochemistry and Biophysics, UCSF, 600 16th St., San Francisco, CA, United States of America; 3 Department of Biochemistry and Biophysics, Center for Cellular Construction, University of California San Francisco, San Francisco, CA, United States of America

**Keywords:** mathematical biology, mitochondria, graph theory, applied topology, persistent homology, statistical mechanics

## Abstract

Mitochondria serve a wide range of functions within cells, most notably via their production of ATP. Although their morphology is commonly described as bean-like, mitochondria often form interconnected networks within cells that exhibit dynamic restructuring through a variety of physical changes. Further, though relationships between form and function in biology are well established, the extant toolkit for understanding mitochondrial morphology is limited. Here, we emphasize new and established methods for quantitatively describing mitochondrial networks, ranging from unweighted graph-theoretic representations to multi-scale approaches from applied topology, in particular persistent homology. We also show fundamental relationships between mitochondrial networks, mathematics, and physics, using ideas of graph planarity and statistical mechanics to better understand the full possible morphological space of mitochondrial network structures. Lastly, we provide suggestions for how examination of mitochondrial network form through the language of mathematics can inform biological understanding, and vice versa.

## 1. Introduction

Cells are highly complex structures that exhibit a wide range of geometrical features on scales ranging from symmetric protein complexes to entire organelles. Although relationships between form and function in biology have been appreciated at a conceptual level for centuries, it was only with the advent of modern mathematics and systematic measurement methods that cell biological systems could begin to be considered as quantitatively-describable entities within a framework that might mechanistically relate their forms and functions. While biology in the twenty-first century has advanced by leaps and bounds in its ability to identify and describe the components that comprise cell biological systems across multiple scales, our understanding of the origins of structures comprising these systems is still in its infancy.

During the scant hundred years since the publication of D’Arcy Thompson’s seminal On Growth and Form, humankind’s ability to observe, capture, and measure biological form has undergone a series of improvements spanning multiple orders of magnitude in both spatial and temporal resolution [1]. From the continued development of every facet of light microscopy, to our rapidly improving capacity to engineer fluorescent labels into living systems, to the creation of computational systems and structures enabling collection of enormous quantities of data, we are presently bombarded on a near-daily basis with new observations of living phenomena. In parallel, mathematics and physics have grown to such a point that the simulation of fluids in three dimensions is routinely possibly using a reasonably inexpensive desktop computer. Recent trends in artificial intelligence, particularly within the development of neural networks, have demonstrated the creation of new tools to measure, evaluate, and compare images at a heretofore-unseen pace in an automated manner.

Yet, for all of the discovery and innovation that has occurred over the past century, there seems to exist a gap between the studies of mathematics and biological form. This is due in large part to a focus of effort on discovering the molecules responsible for biological phenomena, which has proven to be highly productive. We have reached the point now that we are drowning in data, and it becomes more important than ever to start to reach a conceptual understanding of how biological form arises, a question that is inherently mathematical in nature. In line with this reflection should be an awareness that there exists an enormous variety of biological phenomena whose associated forms present rich opportunities for discovery. Here, we focus on one such form and opportunity: the study of mitochondrial networks.

The presence of mitochondria, the ‘powerhouses of our cells,’ is a near-unifying hallmark of eukaryotes—only in 2016 was the first eukaryotic organism (*Monocercomonoides*) discovered that lacked mitochondria [2]. Although there exist subsets and subtypes of eukaryotic cells that lack mitochondria, such as red blood cells, these are by and large exceptions [3]. Indeed, mitochondria are critical for a wider range of cellular functions than just generating ATP as fuel: they enable the critical self-destructive cell apoptosis response, help regulate calcium ion levels in conjunction with the endoplasmic reticulum (ER), generate energy from lipids through fatty-acid metabolism, as well as modulate the reactive oxygen species required by a range of physiological processes including pancreatic function and aging [4–11]. Given the pervasiveness of mitochondrial function in biological regulatory processes, it should not be surprising that mitochondrial dysfunction is associated with a wide range of human diseases, including Alzheimer’s and Parkinson’s disease [12].

Unlike most other organelles, mitochondria cannot be created *de novo*, but instead are derived from growing extant mitochondria. As a result, proper cycles of cellular division typically require the replication of mitochondrial DNA (mtDNA), which independently encodes a small number of proteins critical for mitochondrial function and may also serve other roles, such as innate immune signaling [13–15]. Copies of mtDNA are distributed across the set of all mitochondria in the cell and range in per-cell copy number from ${\approx}5$ in human sperm cells to ${\approx}10^5$ in human oocytes, though they are typically present on the order of 10^2^–10^3^ copies in many human cell types [16–18]. Although not yet fully understood, mtDNA seem to be associated with sites and specific types of mitochondrial fission in combination with ER contacts [19–23].

**Figure 1.**
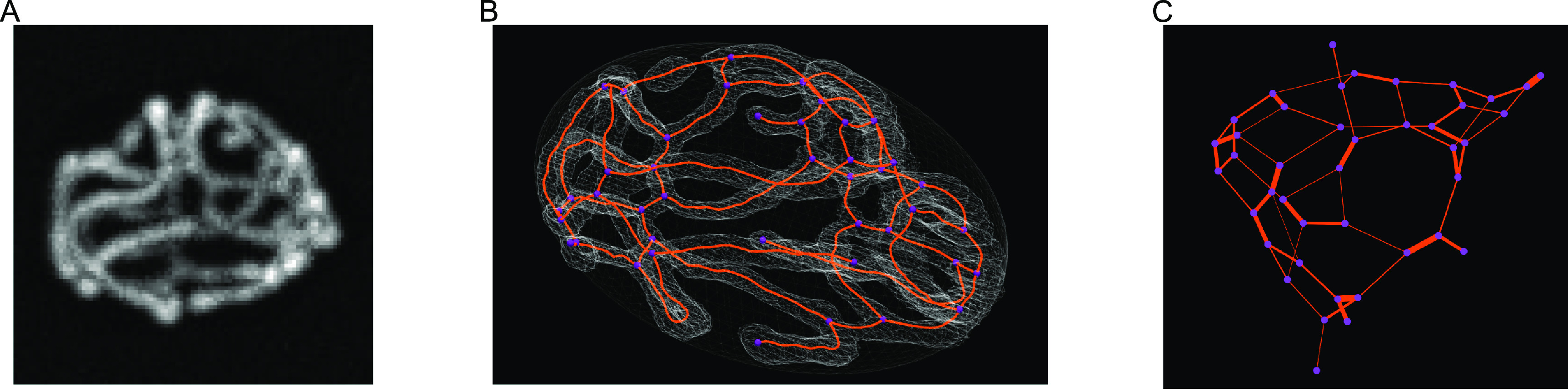
After 3D imaging (A), data from the mitochondrial marker channel is run through MitoGraph, which segments the mitochondria and establishes the graphical skeleton (B), leading to the weighted graphical structure (C). In (C), edges are not drawn to scale—instead, edge thickness denotes the relative mitochondrial tubule length.

Although mitochondria are often depicted as bean-shaped objects, in fact in most cells they form ramifying networks of tubules (figure 1(A)). These tubular networks undergo constant rearrangement, both in structure and position, sometimes in concert with or as a result of interactions with other organelles [19–21, 24–26]. Much is known about the molecular and cellular processes that sculpt these networks [27–37]. However, the biological impacts of mitochondrial network form are not well understood, nor is it clear what determines the specific form of the network in any given cell type.

Why should we care about the structure of mitochondrial networks? A prominent hypothesis for the function of mitochondrial networks is that the combination of membrane potential-dependent fusion and nonspecific fission enables the cell to retain productive and degrade dysfunctional mitochondria [38], suggesting that network structure might reflect overall mitochondrial function. A variety of work has shown that a cell’s mitochondrial network changes structure and dynamics in response to cell cycle progression, environmental perturbations, and cell state [39–41]. The extremes of eliminating mitochondrial fission and fusion lead to substantial changes in cellular physiology and fetal development [42]. Apoptosis is associated with mitochondrial fragmentation and swelling, while increased mitochondrial fusion in budding yeast may increase their replicative lifespan [43, 44]. Diffusive transport within networks is more efficient when loops are present, as is typically the case for wild-type mitochondria [45, 46]. Broadly speaking, it seems that major changes to mitochondrial morphology are associated with an enormous variety of altered cellular states.

To date, it remains unknown how mitochondrial dynamics induce or reflect changes in cellular state; by and large, only extreme and irreversible endpoints have been studied [47–50]. As a result, it seems that a better understanding of how mitochondrial dynamics are associated with transitions through cellular state space would at least provide a non-terminal imaging-based means of tracking such transitions and may even inform their nature. In order to describe the mitochondrial dynamics, we must have a way to quantitatively describe mitochondrial structure and morphological changes; additionally, such a quantitative description may provide a useful state space in which to track changes in cellular state. Using graph theory, topology, and mass-action-like kinetics, we provide a range of approaches to quantitatively describing mitochondrial structure and morphological changes.

In this article, we will focus on mitochondria of the budding yeast, *Saccharomyces cerevisiae* (*S. cerevisiae*). We focus on this organism for several reasons. First, yeast mitochondria are best understood in terms of genetics and molecular biology, with a host of known mutations that alter mitochondria morphology and function [51–60]. Second, the small size of yeast cells has enabled the complete mitochondrial network structure to be determined in individual cells, something that is far more difficult for larger mammalian cells [47, 48, 61–63]. Finally, and most importantly for our present purposes, the mitochondria of budding yeast are confined to the surface of the cell, which allows important constraints on the mathematical representation of their structure [64, 65].

Generally, the shape of an isolated mitochondrion exists on a spectrum ranging from nearly-spheres to tubules. These forms reflect the physical structures comprising and associated with mitochondria. As organelles bound by a lipid bilayer and containing fluid, small and isolated mitochondria typically revert to a minimal-energy spherical shape [42]. However, when bound to the ER, the plasma membrane, and/or the rigid rod-like structural proteins within a cell, individual mitochondria often take on a tubular form. The movement of bound molecular motors or other bound proteins can induce structural and corresponding morphological changes in mitochondria: this can take the form of either a change in tubule length or the drawing-out of a branching tubule from an existing mitochondrion (termed here as ‘outgrowth’).

Although mitochondria can be quantitatively characterized by a number of possible morphological descriptors, the fact that mitochondria are so often present in the form of interconnected tubules suggests that networks are natural descriptors of their overall shape and structure [66, 67]. In fact, the structure of such a network is mandated by a combination of physics (via membrane strain minimization) and mathematics (via graph theory).

**Figure 2.**
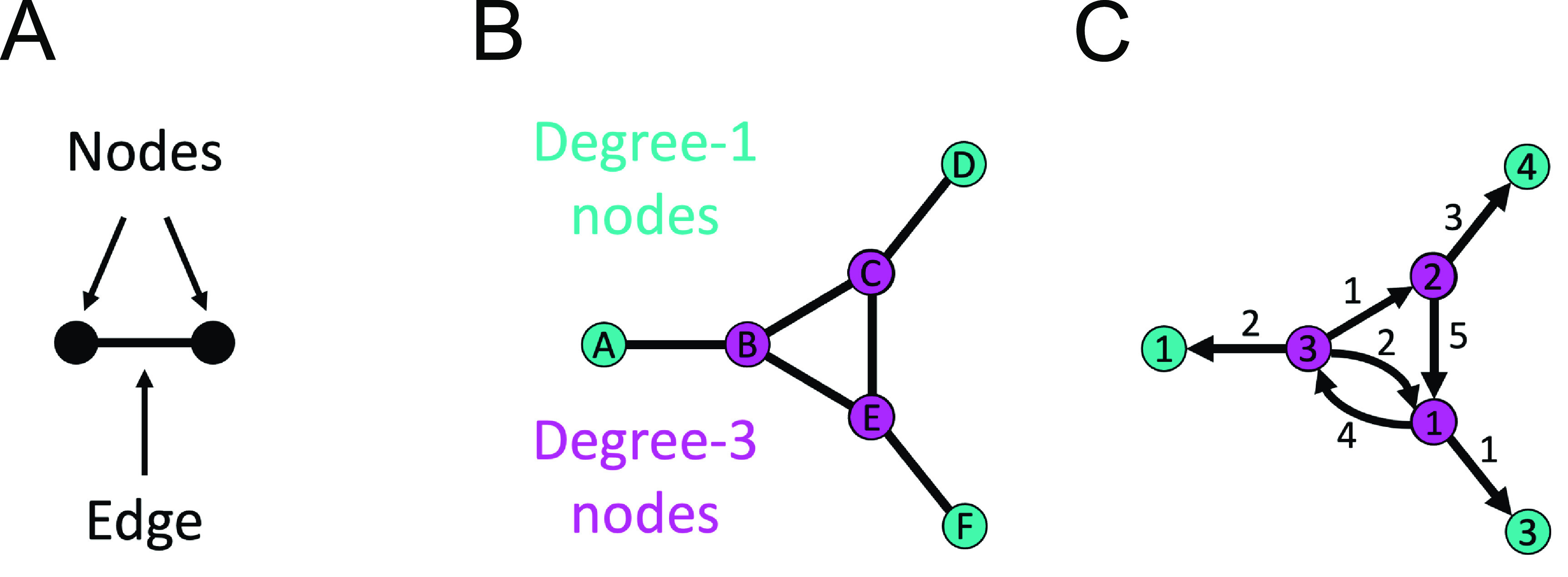
(A) Graphs are mathematical objects made up of points (nodes, vertices) and lines (edges) connecting none, some, or all possible pairs of nodes. (B) The degree of a node in an unweighted graph is the number of edges emanating from that node: teal nodes are degree-1 (nodes A, D, and F) while magenta nodes are degree-3 (nodes B, C, and E). (C) Nodes and edges can have weights that correspond to useful quantities, such as edge weights representing the cost to travel between two city-nodes and node weights representing the cost of staying in that city. Edges can be undirected (no arrow) or directed (arrow), with travel from one node to another allowed if and only if there is an edge beginning at the first node and ending (arrowhead) at the second node. Cycles are paths through a graph that start and end at the same node (e.g. the loop formed by the magenta nodes and their edges to one another). The number of connected components of a graph is the number of disconnected islands of the entire graph: a graph consisting of two copies of the graph in (B) would have two connected components.

If we represent the backbone of the mitochondrial network as a mathematical graph (figure 2), where we assign nodes (points, vertices) at the centers of *Y*-junctions and the ends of tubules, then join those nodes by edges (lines) if they are connected by a continuous tubule, we can immediately learn some network constraints.

Nodes of degree 1, corresponding to ends of tubules, are permitted. Two-way tubule junctions do not have a clear biological interpretation; Sukhorukov *et al* describe them abstractly as regularly-spaced locations along mitochondria where fusion can occur [68]. We see three-way *Y*-junctions in nearly every experiment, corresponding to nodes of degree 3. Four-way *X*-junctions, however, are rarely encountered in budding yeast mitochondrial networks (as opposed to e.g. 3D networks in cardiomyocytes). This may be due to the configuration being topologically unstable, such that a small perturbation at the four-way *X* junction can quickly resolve to two stable *Y*-junctions (a ‘T1 elementary topological transition’), or due to differences in the Helfrich energies of the configures favoring two *Y*-junctions [69, 70]. Following the same reasoning, we suggest that junctions defined where mitochondrial tubules are fused will only transiently have a degree greater than 3.

Mathematics provides additional constraints. The handshaking lemma states that, if we add up the number of edges emanating from each vertex (the degree of each vertex), it equals twice the total number of edges in the graph,

\begin{equation*} \sum_{v_{i} \in V(G)} \textrm{deg}(v_{i}) = 2 |E|, \end{equation*}

where *V*(*G*) is the set of nodes of our graph *G* and $|E|$ is the total number of edges in *G* [71]. Since $2|E|$ is an even number, and deg$(v_{i})$ is always odd (the end of a tubule has a single edge emanating from it, and a three-way junction has three), there must necessarily be an even number of nodes in the graph.

We can go a step further: the Euler characteristic of a surface *χ*, a topological property of surfaces that depends on the number of holes or handles on the surface, is equal to the number of vertices ($|V|$), minus the number of edges ($|E|$), plus the number of faces ($|F|$), minus the number of connected components ($C \geqslant 1$) for a graph embedded on that surface [71, 72]. Because the network incompletely covers the sphere, the space it occupies is equivalent to a standard 2D plane, inducing a planar graph restriction on mitochondrial networks. In budding yeast, the mitochondrial network sits underneath the surface of a spheroid, and for such a closed surface, *χ* = 2. This leads to a relationship between $|F|$, $|E|$, and $|V|$ for the mitochondrial networks. Each face of our graph consists of at least three edges, and each edge can be shared by at most two faces. Therefore,

\begin{align*} 3 F \leqslant 2 |E|\\ |V| - |E| + |F| - C = 1. \end{align*}

Combining these inequalities leads to (2), providing an upper bound for the number of edges in a mitochondrial network in terms of the number of vertices and connected components:

\begin{align*} |E| \leqslant 3 |V| - 3 - 3C \leqslant 3 |V| - 6. \end{align*}

This result, which follows from Euler’s formula for planar graphs, gives us yet another constraint on the structure of the mitochondrial network. By representing the network with a mathematical structure, we can rapidly limit the space of possible mitochondrial network structures that we have to consider inside budding yeast. The Universe of networks is further constrained via Kuratowski’s theorem, which describes particular structures prohibited in the planar networks we consider here [71].

In many organisms, proteins exist and function to induce physical alteration of mitochondrial structure, both within a single tubule and across multiple tubules. The most well-known of these processes are fission, fusion, and mitophagy. Fission describes the division of a mitochondrial tubule, though not necessarily into two isolated mitochondria. Fusion describes the opposite process: the joining of two mitochondrial tubules to become a single unit. Mitophagy, or the autophagy of mitochondria, is a cellular process for degrading mitochondria, likely to remove dysfunctional entities from the cell. In addition to the processes described above, mitochondrial morphology may also be altered through ‘resorption’ (the absorption of a terminal or pendant mitochondrial branch into its adjoining tubule), though this process is poorly characterized; its inverse process is ‘outgrowth,’ described above [64].

Through a combination of the mitochondrial structural processes described so far, an enormous range of mitochondrial superstructures can be constructed, and indeed found, within cells. Such mitochondrial networks are readily visible through a high-power light microscope; under observation, these networks can be seen to undergo both large-scale motion and reconfiguration, demonstrating that the mitochondrial network of a cell is a dynamic system. This knowledge is not new; indeed, there are reports of mitochondrial networks published in the late 1800s [73, 74]. What remains unclear is what determines network morphology for mitochondria, in particular, are local processes like fission and fusion, taking place at random, sufficient to explain the networks that we see, or does the cell need to actively monitor and adjust its mitochondrial networks? Answering this type of question requires a way to predict the types of networks that a given set of morphological processes should produce, their statistical distribution, and the effect of mutations on the network organization. This, in turn, requires a conceptual framework for thinking about mitochondrial structures and dynamics that can bridge the gap between small scale local processes like fission/fusion and large scale properties like network connectivity. Graph theory provides such a framework, and our goal here is to explore the implications of graph theory results for mitochondrial structure, and vice versa.

## 2. Motivation for this work

### 2.1. How can studying mathematics and physics inform our understanding of mitochondrial networks?

In order to understand the relationship between mitochondrial network form and function, we must have a way of quantifying each aspect. As networks, mathematics and physics provide tools and representations that can and have been used for the quantification of mitochondrial network form. These tools and representations provide more than just a means of describing the networks, however; prior work in physics and mathematics can inform our understanding of physiological steady states (via the Perron–Frobenius theorem), ergodicity (through state-space representation of networks), and variation (using global and local spatiotemporal measures) in mitochondrial networks [75, 76].

Graph theory and topology offer some of the most useful tools and representations for quantifying and comparing mitochondrial networks.

Using graph theory, we can represent mitochondrial networks at increasing levels of complexity, from unitless geometric representations at one end to spatially embedded and constrained tubules at the other. It also provides tools for deriving unique representations of individual networks, as well as local and global metrics for describing different features of those networks [71].

Tools from applied topology, while not typically used in the consideration of mitochondrial networks, provide representations for describing networks and their essential properties across multiple length scales [77]. Of these representations, we will in this work focus on the application of ‘persistent homology’ and its associated metrics, which provide broadly applicable and easily computable ‘barcodes’ that themselves can be compared with their distances measured in an informative manner [78, 79].

Additionally, following up on questions raised in the previous subsection, a better understanding of the mathematical structure underlying the space of ‘mitochondrial’ graphs may give additional insight into what we see under the microscope. How much do mitochondrial networks vary across a population of cells? How similarly do networks evolve in different cells? Are there steady-state distributions of mitochondrial network structures we expect to see in large populations of cells, and if so, how do they compare to networks seen over time in a single cell (i.e. is network evolution an ergodic process)?

The remainder of this work is organized as follows. Firstly, we will review some of the extant literature, give an overview of outstanding questions regarding mitochondrial network morphology and dynamics, and briefly describe relevant experimental methods. Secondly, we will explore mathematical and physical questions raised by the study of mitochondrial networks, emphasizing methods of representation and measurement, as well as provide a link to state spaces and their associated statistical mechanics. Lastly, we will describe some biological questions raised by the study of mitochondrial networks through the lenses of mathematics and physics, with an aim of describing potential links between network morphology and cellular function.

### 2.2. Why is it now an opportune time to heavily study the intersection between mathematics and mitochondrial networks?

Firstly, advances in experimental techniques, in particular wider-spread adoption of microscopy with high spatiotemporal resolution (e.g. iSIM and lattice light-sheet), enable us to capture mitochondrial network dynamics on an event-by-event basis for longer periods of time than ever before [63, 80–85]. Secondly, the low cost of computational infrastructure makes storage, processing, and analysis of such data not just possible but reasonable; additionally, the same infrastructure can be used to simulate mitochondrial networks *in silico*, accelerating the experiment-theory feedback cycle and allowing it to be implemented within a single laboratory [86–91]. Lastly, recent widespread adoption of so-called ‘black box’ machine learning techniques have catalyzed a corresponding movement for understanding biological systems using interpretable theory-based approaches, and the intersection of mathematics and mitochondrial networks provides opportunities for study using both classes of techniques, along with the potential to build bridges between them.

### 2.3. How can studying mitochondrial networks benefit mathematics and physics?

Networks that change over time are the subject of study across a wide range of fields, from the study of social interactions networks in sociology, to neuronal rewiring within brains in neuroscience, and beyond [92–94]. However, our understanding of the ‘rules’ guiding the evolution of those networks is still quite narrow.

How can we develop better theory and tools for the investigation of complex, evolving networks? Ideally, we would test new hypotheses on real-world datasets of an addressable size that change in limited, understandable ways. Mitochondrial networks satisfy both of these criteria: the number of nodes and edges is computationally addressable (on the order of hundreds or fewer in *S. cerevisiae*) and there exist fewer than ten types of structural modifications that can occur within the network [48]. Also, the morphological changes that take place on mitochondrial networks, in combination with the rules limiting network structure, form a heretofore uncharacterized class of graphs: planar graphs exclusively of degrees 1 and 3. Having a biologically-inspired generative mechanism for these graphs complements the traditional combinatorics-based approaches to characterizing graph classes.

These considerations immediately lead to further questions, What is the space or range of network structures, i.e. graphs, that mitochondria take on inside living cells? How does the space and variety of mitochondrial network structures differ from that of mitochondrial-like networks? What is the structure of mitochondrial network space under the restriction of ‘biological’ graph modifications?

### 2.4. What is the current state of the field?

Although a great deal of research has been done on mitochondrial structure and function, we will limit our brief review of the literature to prior work that has included efforts at quantitatively modeling the mitochondrial network. These studies are not large in number. Susanne Rafelski and collaborators developed the segmentation and analysis software MitoGraph (figure 1), which has enabled them to show (among other results) that mitochondrial network volume scales linearly with cellular volume, that mitochondrial networks resemble ‘geographical networks,’ and the fission–fusion double knockouts exhibit quantitatively distinct network properties from those in wild-type yeast [47, 48, 61]. Meyer-Hermann *et al* developed a graph-theoretic algebraic framework for describing mitochondrial networks and their relationship with the mammalian cytoskeleton [68, 95]. Shirihai *et al* have conducted studies on the relationship between mitochondrial dynamics and quality control, as well as how those concepts might be related to network structure [96, 97]. Johnston *et al* have published many papers pertaining to mtDNA population dynamics; some of their more recent publications examine possible benefits of mitochondrial network structure from the perspectives of both cellular physiology and mitochondrial mutational burdens [38, 98–101]. Chialvo *et al* combine the theoretical framework from Meyer-Hermann’s group and progressive coarse-graining of mitochondrial images to study mitochondrial network structures through the lenses of criticality and percolation theory [49, 50]. Quinn *et al* use a range of representations of mitochondrial networks, such as dynamic social networks and embeddings derived from graph convolutional neural networks, to evaluate changes in mitochondrial morphology over time [102–105]. The Mitometer algorithm from Lefebvre *et al* uses graph features to help track the motion of mitochondria [91].

## 3. Mathematical questions raised by the study of mitochondrial networks

We begin with some definitions.

Networks are often represented as graphs (figure 2): mathematical structures consisting of points (nodes, vertices) that may be connected to one another by lines (edges) (figure 2(A)). The degree of a node counts the number of edges emanating from that node (figure 2(B)). Edges may be assigned weights (numerical values) describing some characteristic of that edge (e.g. tubule length, distance between rail hubs, pipe diameters, etc) (figure 2(C)). Graphs with weighted edges are known as ‘weighted graphs’ and those lacking weights as ‘unweighted graphs.’ Weighted graphs may be embedded into spaces whose axes have the same units as the edge weights; we call these ‘spatially embedded graphs.’ Increasing the information associated with the representation of a network (e.g. weights, locations) correspondingly increases the complexity of analysis. For that reason, we focus here mainly on unweighted graphs, though we do not mean to imply that weighted or spatially-embedded graph representations have no role in representing further aspects of mitochondrial networks [71].

### 3.1. Mitochondrial networks as unweighted graphs

In wild-type *S. cerevisiae*, mitochondria are located beneath the spheroidal cell cortex, inducing mitochondrial networks to be planar (due to the concordance between the surface of a two-dimensional sphere and the two-dimensional Euclidean plane joined with a point at infinity) [48]. We note that the planarity claim above may not hold in some cell types due to substantial differences in cytoskeletal structures, mitochondrial tethering, and other biological features, but in budding yeast it is physically enforced by the tethering of mitochondria to the cell cortex [65].

Graph representations of mitochondrial networks by both Sukhorukov and Rafelski involve assigning nodes to three-way junctions of tubules and ends of tubules, then inserting an edge between two nodes when they are physically connected by a continuous membrane [47, 48, 68, 95]. As a result, we can classify mitochondrial networks as undirected, unweighted, simple (no edges from a node to itself) planar graphs with nodes of degrees 1 or 3 that are not necessarily connected. To our knowledge, there does not exist a specific classification in mathematics corresponding to this set of graphs, and we therefore refer to it here as the set of ‘mitochondria-like graphs.’ However, a well-studied class of graphs forming a subset of the mitochondria-like networks are cubic graphs, which are constructed exclusively from degree-3 nodes. Although much is known about cubic graphs, the incorporation of pendant (degree-1) nodes into such graphs allows for the possibility of boron tree-like mitochondrial networks whose structures correspond to boron tree graphs (unrooted binary trees with nodes of degrees 1 and/or 3) [106]. These structures may also emanate from edges of cubic graphs or serve as bridges between them, though not all such constructions are allowed. As a result, the space of possible mitochondrial graph structures is substantially greater in size than the numbers of cubic graphs and boron trees for a given number of nodes. Another related structure is the Bethe lattice, consisting of single loop-free component with infinite nodes all of which are degree three, which can be seen as an infinite limit of the boron tree. Viana *et al* showed that Bethe lattices exhibit features similar to fission-fusion double-knockout mitochondrial networks, likely due to those mutants’ typically tree-like mitochondrial networks [48]. At the other end of the spectrum, Brown *et al* modeled particle search times for intact and ‘decimated’ honeycomb networks, demonstrating that increasing the number of loops in such networks lead to continual improvements in efficiency [45].

Given this set, we can construct a corresponding state space (‘mitochondrial network state space’) by assigning different (non-isomorphic) graphs as unique states and allowing transitions between two states if a single morphological change (fission, fusion, etc) performed on the first graph induces an isomorphism between the modified and second states. Our choices of an unweighted-graph representation and isomorphism-based difference are not unique; we discuss additional representations later in the article.

Having selected a quantitative representation of mitochondrial network structures, we are now confronted with the problem of how to compare those structures. The binary evaluation of whether two mitochondrial network structures are identical is known in mathematics as the graph isomorphism problem. Although in general the time complexity of evaluating whether two graphs are isomorphic is now believed to be quasi-polynomial, planarity reduces the time to the square of the number of vertices or less [107, 108]. Planar graphs can be given unique identifiers (canonized) that have length in the order of the logarithm of the number of vertices and efficient software exists to generate these canonizations [109]. The ability to index all possible mitochondria-like graphs also provides a framework for counting the distribution of graphs in real datasets. Although great progress has been made in the development of planar graph algorithms, many basic questions are still unanswered.

Given these definitions and background, we can now ask deeper-reaching questions. Some of these questions are primarily mathematical in nature. For example, how many mitochondria-like graphs are there, for a given number of nodes? As with the general case of enumerating unlabeled planar graphs, an analytical expression to calculate this number has not yet been reported. Upper and lower bounds can be established, however.

The planarity of mitochondria-like graphs enables us to use extant results from the full family of planar graphs to set a loose upper bound of $30.061 ^ {N}$, which was derived using information-theoretic methods [110].

For a lower bound, we can consider the sum of two classes of graphs. The first is the set of unlabeled cubic outerplanar graphs, which only have nodes of degree 3 and can always be drawn in a 2D plane such that all nodes are adjacent to the outermost face of the graph. For example, a pyramid or tetrahedron with nodes assigned to its corners is not outerplanar when flattened onto the plane because it must always contain a node drawn within the boundary of that drawing. We use the size of this set as a lower bound because there does not presently exist an asymptotic scaling relation for unlabeled cubic planar graphs [111]. The second component of the lower limit is the set of ‘boron trees,’ which are trees with nodes of degree either one or three [112, 113].

Combining these bounds gives the following expression:

\begin{align*} &amp;\frac{1.255 * (2.48)^{|V|}}{|V|^{2.5}} + \frac{0.009099 * (7.5036)^{|V|}}{|V|^{2.5}} \nonumber\\ &amp;\quad \leqslant |M_{G}(|V|)| \lt 30.061^{|V|}, \end{align*}

where $|M_{G}(|V|)|$ is the number of mitochondria-like graphs with $|V|$ vertices. These bounds are loose, as they were obtained for simple connected graphs, while mitochondria-like graphs may be composed of multiple connected components that may not belong to the classes of cubic outerplanar graphs or boron trees.

It is possible to outline a recursive procedure for determining the number of mitochondria-like graphs for a given number of nodes. We reiterate that mitochondrial-like networks are not necessarily connected in a single component: at any given point in time, there is no guarantee that every node or edge is accessible to all of the others. Therefore, to calculate the number of unique (non-isomorphic) mitochondria-like graphs, we can use the following relation:

\begin{align*} |M_{G}(|V|)| = \sum_{K \in p(|V|)} \prod_{k \in K} \left(\begin{array}{c}|M_{G}(k)| + m(k,K) - 1\\ m(k,K)\end{array}\right) , \end{align*}

where $p(|V|)$ is the number of partitions of $|V|$, which enumerates the unique ways to generate the positive integer $|V|$ from positive integers; *K* is a partition of $|V|$ unique up to permutation; *k* is a member of that partitioning; and $m(k, K)$ is the multiplicity of *k* in *K* (that is, how many times *k* appears in *K*).

Conceptually, we can interpret (3) as follows. A mitochondria-like graph with $|V|$ nodes may consist of a single connected component containing $|V|$ nodes, two connected components (separated networks) containing $|V| - k$ and *k* nodes, three connected components containing $|V| - j - k$ and *j* and *k* nodes, etc. If we add up the number of mitochondria-like graphs that contain each of the smaller number of nodes and add that to the number of mitochondria-like graphs that consist of a single connected component containing N nodes, we should end up with the total number of possible mitochondria-like graphs. However, some partitions may include multiples of the same number (e.g. $4 = 1 + 1 + 2$, with 1 showing up twice), and so we must account for identical smaller networks appearing in duplicate (requiring calculation of combinations with replacement).

**Figure 3.**
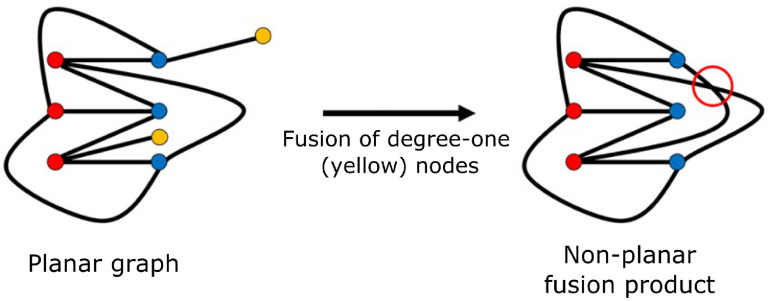
Theoretical fusion event leading to non-planar graph. Mitochondrial-like networks must be planar, so the depicted fusion event is not biologically permitted, but is still mathematically possible, requiring additional constraints on fusion processes. Nodes represent physical three-way junctions and ends of mitochondrial tubules; edges signify a mitochondrial tubule connecting the two nodes. Yellow nodes from the planar graph on the left undergo fusion to form the non-planar graph on the right due to an unresolvable edge crossing. It is not possible to draw the resulting graph in a plane such that each red node has edges to each blue node.

Carrying out this computation is non-trivial: although the recursive definition allows previously stored results to be re-used, the process of generating all connected and non-isomorphic mitochondria-like graphs for a given number of nodes must be done exhaustively. Although we would like to take inspiration from the underlying biological processes, any strategy for doing so must take into account the constraints on morphology of mitochondria-like graphs in terms of planarity and degree. For example, it must be noted that a theoretical ‘fusion’ event can lead to the generation of a non-planar graph from an originally planar mitochondria-like graph (figure 3).

This observation immediately leads to additional mathematical questions. Under what morphological operations (e.g. fission, fusion, etc) do graphs form closed sets? Can we define a fusion operation that is guaranteed to result in a mitochondria-like graph, and if so, how? One possible solution for defining a fusion process that respects biological constraints such as planarity is to represent graphs embedded in space, replacing operations on graphs with operations on projections. Some considerations related to embedding mitochondria-like graphs in space will be discussed below. Does such a fusion function, in combination with the other morphological operations, enable the generation of all mitochondrial-like networks? What features, measures, or heuristics of these graphs exhibit the most variance or are most informative?

Before moving on, we believe it worth noting that the graph-based description of mitochondria, while generally effective, is not necessarily comprehensive. For instance, donut-shaped mitochondria with toroidal skeletons have been reported in the literature as a result of cellular stress (e.g. hypoxia) and can be generated through the fusion of the two ends of an isolated rod-like mitochondrion or the fusion of two rod-like mitochondria, but our formulation does not permit such a structure [41, 114–117]. Using more generalized representations, such as pseudographs that allows loops and multiple edges between the same pair of nodes or hypergraphs, may more effectively and completely describe mitochondrial network structure [118–122]. At the time of writing, however, relatively few computational tools exist to conduct analyses using those representations.

### 3.2. Statistical mechanics of mitochondrial network space

In the preceding section we described how to enumerate the possible mitochondria-like networks that could exist subject to the constraints on graph structure. This enumeration leads to the question of which mitochondria-like graphs can actually be produced by known physical processes that operate on real mitochondria. Given the processes that sculpt the mitochondrial network, can one reach any network in the space of possible networks, starting from any other network? Is the answer to the prior question different if those processes occur randomly in space and time? In other words, are all mitochondria-like graphs reachable starting from any particular graph? Second, can we predict the distribution of mitochondria-like graphs that should be observed at steady-state? The first question falls into the realm of evolutionary graph theory while the second represents statistical mechanics.

Given the unweighted graph representation of mitochondrial networks, we can explicitly construct and define mitochondrial network state space.

We first define the states. Here, mitochondrial networks will be designated as different states if their unweighted graph representations are non-isomorphic. Two states are accessible (adjacent) if there is a single morphological change that can convert one state into the other.

**Figure 4.**
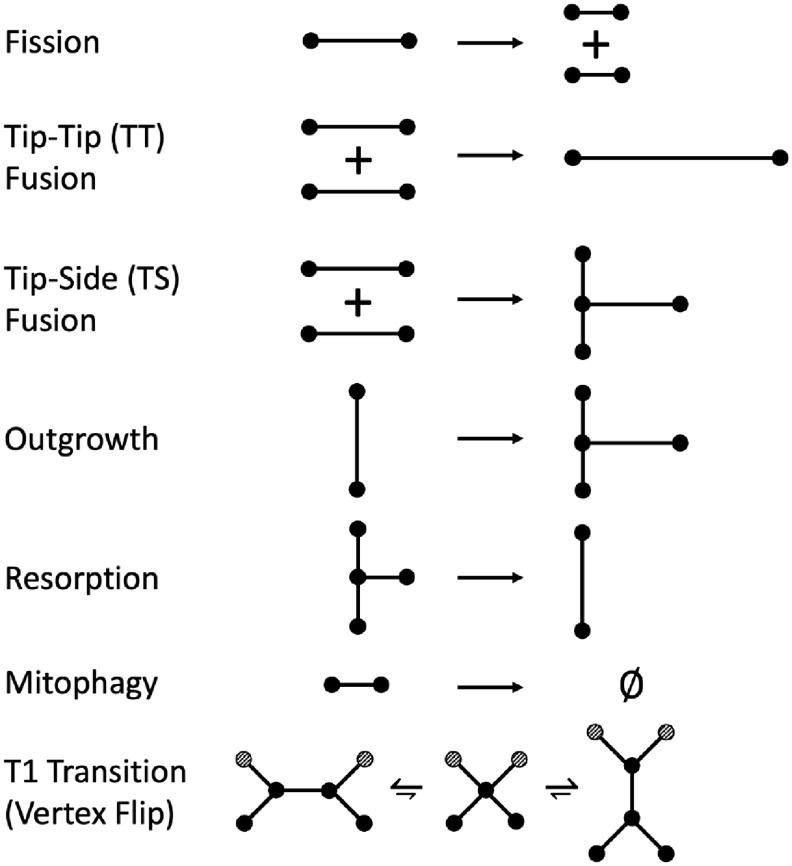
Morphological operations in mitochondrial networks.

Mitochondrial networks are known to undergo seven types of morphological changes, with the results of changes to minimal relevant structural elements (e.g. a single edge, component, etc) illustrated in figure 4. In depicting these local operations, we assume that in a single step only the shown nodes and edges can undergo a change. The possible operations are:

(i) Fission
  (a) Type 1: Fission maintains the number of connected components
  (b) Type 2: Fission breaks a component into two new connected components
(ii) Tip-tip Fusion (TT-fusion)
  (a) Type 1: Fusion maintains the number of connected components
  (b) Type 2: Fusion joins two connected components into one new connected component
(iii) Tip-side Fusion (TS-fusion)
  (a) Type 1: Fusion maintains the number of connected components
  (b) Type 2: Fusion joins two connected components into one new connected component
(iv) Outgrowth: a new tubule emerges from the side of an existing tubule
(v) Resorption: an extant tubule is absorbed into one of its adjacent tubules
(vi) Mitophagy: a network component is degraded
(vii) Vertex Flip: two adjacent degree-3 nodes transiently merge and re-equilibrate in a flipped orientation.

How do these changes relate to one another? Firstly, we note that fission and TT-fusion are inverse operations, as are outgrowth and resorption. Mitophagy does not have a single-operation inverse, as there is no *de novo* mitochondrial biogenesis in the absence of extant mitochondria, though fission on isolated two-node components may serve as a functional inverse. Additionally, we assume that only isolated mitochondrial tubules, consisting of two degree-1 nodes connected by an edge, are subject to mitophagy and thereby destroyed. In order to account for this, we introduce the dispersion parameter *α*, defined as the fraction of degree-1 nodes that are adjacent to other degree-1 nodes. It is unclear whether TS-fusion has a single-operation inverse: it is possible that fission could take place at a three-way junction, but there is not yet biological evidence to support this as a sufficiently common occurrence; alternatively, in the case of pendant edges, resorption could serve as a specialized inverse operation. Vertex flips alter the local configuration of a mitochondrial network but do not change the number of each type of node, enabling more rapid exploration of graph structures.

Equipped with states and permissible transitions, we can represent mitochondrial network state space itself as a graph: the nodes are non-isomorphic mitochondrial-like networks and an edge is constructed between a pair of nodes if one of the above morphological operations converts one node into the other. A standard unweighted graph may not be the best representation of this space, however: it is reasonable to associate directionality with state transitions, as morphological operations are not their own inverses, suggesting a directed-graph state space representation. Similarly, there may be multiple morphological operations that convert one state into another, in which case a choice must be made whether to aggregate equivalent operations together, or to allow multiple (potentially directed) edges in the structure (enabled via a hypergraph, multigraph, or pseudograph structure). Lastly, assigning edge weights in state space can be useful: these could correspond to the multiplicity of morphological operations (i.e. fission on distinct edges leading to the same resultant graph), experimentally measured transition rates, and/or other relevant properties.

Construction of this state space leads to further questions, some of which are more biological in nature. What are the fundamental properties of mitochondrial network state space? Does the state space exhibit some form of detailed balance? Are the dynamics of mitochondrial network morphology ergodic? How does the set or space of mitochondrial networks that cells ‘sample’ compare to the space of mitochondria-like graphs? If two or more cells exhibit the same mitochondrial network ‘state’ at some point in time, how do the future trajectories of those cells through mitochondrial network state-space differ? If the observed distribution of graphs is substantially different from that predicted by the model, it would suggest that the model is missing one or more additional processes or else is missing regulatory linkages governing the processes: for example, a dependence of one or more of the rates on one or more structural features of the graph.

We can begin to address these questions through the development of mass-action-like equations describing the change in the number of different node types over time, with *n*_1_ and *n*_3_ corresponding to the number of nodes of degrees 1 and 3 in the network, respectively. Although the number of each node type is a non-negative integer, we can formulate equations in the continuum limit using a chemical-reaction-theory-like approach for the purposes of steady-state analysis.

We make two sets of assumptions. First, that TT- and TS-fusion are both second order processes, as nodes require other nodes or edges for fusion. Second, that fission, mitophagy, outgrowth, and retraction are all at most first-order, as these events do not seem to require interaction between multiple nodes but may depend on the overall size of the network. These lead to rate equations of the following form (though the exact terms may differ):

\begin{align*} \textrm{Fission }\, \beta_{\textrm{fis,1}} = 2 k_{\textrm{fis}} |E| = k_{\textrm{fis}} \left ( n_{1} + 3 n_{3} \right ). \end{align*}

\begin{align*} &amp; \textrm{TT-Fusion }\, \beta_{\textrm{ttf,1}}\nonumber\\ &amp; \quad = -\frac{1}{2} k_{\textrm{fus,tt}} \Bigg [ \alpha n_{1} \left ( \alpha n_{1} - 2 \right ) + 2 \alpha \left ( 1 - \alpha \right )n_{1}^{2} \phantom{\beta_{\textrm{ttf,1}} } \phantom{=} \nonumber\\ &amp; \qquad + \frac{1}{2}\sum_{v_{i} \in L(G)} \bigg [ 2 ( 1 - \alpha ) n_{1} - 2 \nonumber\\ &amp; \qquad - \sum_{v_{j} \in \{N_{2} ( v_{i} ) \cup N_{3} ( v_{i} )\}} {\Big ( 3 - \textrm{deg}(v_{j}) \Big )} \bigg ] \Bigg ]. \end{align*}

\begin{align*} &amp;\textrm{TS-Fusion }\, \beta_{\textrm{tsf,1}} \nonumber\\ &amp;\;\; = -k_{\textrm{fus,ts}} \Bigg [ \alpha n_{1} \bigg ( |E| - 1 \bigg ) + \big (1 - \alpha \big ) n_{1} \bigg (|E| - 3 \bigg ) \Bigg ] \nonumber \\ \phantom{\beta_{\textrm{tsf,1}}} &amp;\;\; =-\frac{1}{2} k_{\textrm{fus,ts}} n_{1} \left [ n_{1} + 3n_{3} + 4 \alpha - 6 \right ]. \end{align*}

\begin{align*} \beta_{\textrm{tsf,3}} &amp;= -\beta_{\textrm{tsf,1}} \nonumber \\ \textrm{Outgrowth } \beta_{\textrm{out,1}} &amp; = \beta_{\textrm{out,3}} = k_{\textrm{out}}|E| \nonumber\\ &amp; = \frac{1}{2} k_{\textrm{out}} \left( n_{1} + 3n_{3} \right).\qquad \end{align*}

\begin{align*} \quad\!\textrm{Resorption } \beta_{\textrm{res,1}} &amp;= \beta_{\textrm{res,3}} = -k_{\textrm{res}}(1 - \alpha) n_{1}. \end{align*}

\begin{align*} \textrm{Mitophagy } \beta_{\textrm{aut,1}} &amp; = - 2 k_{\textrm{aut}} \alpha.\qquad\qquad\quad \end{align*}

Here, *α* is the dispersion fraction defined above and $\beta_{X,Y}$ represents the rate of process *X* acting on nodes of degree *Y*. In the formula for TT-Fusion (5), *L*(*G*) refers to the leaves (degree-1 nodes) of the graph *G* and $N_{k} (v_{i})$ is the set of nodes in the *k*-neighborhood of *v*_*i*_ (the set of nodes accessible after *k* hops). The sum over neighborhoods is performed in order to avoid prohibited fusion events within each connected component, which lead to self-loops or multiple edges between the same pair of nodes. We describe mitophagy as occurring at a constant rate, though it can only affect the relevant sub-population of degree-1 nodes. The rate constants *k*_*i*_ are experimentally determined. We must note that these equations are unlikely to describe the full behavior of geometrically-constrained mitochondrial networks, as the breadth of fusion events permitted by this theoretical framework can allow tubules to fuse that would not be able to do so *in situ*, leading a prohibited network structure due to a loss of graph planarity.

Combining rates for *n*_1_ and *n*_3_ enables us to capture the net rate of creation and destruction of those node types, leading to rate equations of the following form:

\begin{align*} \frac{\mathrm{d} n_{1}}{\mathrm{d} t} &amp;= \beta_{\textrm{fis,1}} + \beta_{\textrm{ttf,1}} + \beta_{\textrm{tsf,1}} + \beta_{\textrm{out,1}} + \beta_{\textrm{res,1}} + \beta_{\textrm{aut,1}} \end{align*}

and

\begin{equation*} \frac{\mathrm{d} n_{3}}{\mathrm{d} t} = \beta_{\textrm{tsf,3}} + \beta_{\textrm{out,3}} + \beta_{\textrm{res,3}}. \end{equation*}

Taking the steady-state limit of $\frac{\mathrm{d} n_{1}}{\mathrm{d} t} = \frac{\mathrm{d} n_{3}}{\mathrm{d} t} = 0$ for (10) and (11) leads to:

\begin{equation*} \beta_{\textrm{fis,1}} + \beta_{\textrm{aut,1}} = 2\beta_{\textrm{tsf,3}} - \beta_{\textrm{ttf,1}}. \end{equation*}

By invoking our prior assumptions about the reaction orders of mitochondrial reconfiguration processes, we can rewrite (12) in terms of *n*_1_ and *n*_3_ to form the most general model describing those processes:

\begin{equation*} a_{0}n_{1} + a_{1}n_{3} + a_{2} = b_{0} n_{1} ^{2} + b_{1} n_{1} n_{3} + b_{2} n_{1} \end{equation*}

Here, the parameters *a*_*i*_ and *b*_*j*_ are linear combinations of rate constants from (4)–(9). We exclude a first order term in *n*_3_ on the right-hand side (RHS) of (13) as it would require tip-to-side fusion to increase in probability if the number of edges increases, regardless of the number of degree-1 nodes, while both types of nodes are required to execute the fusion operation. Similarly, we also exclude a constant term from the RHS of (13) as tip-to-side fusion is impossible for the many states lacking degree-1 nodes, prohibiting a constant baseline probability of fusion.

Rearranging (13) leads to an expression for *n*_3_ in terms of *n*_1_ at steady state:

\begin{equation*} n_{3} = \frac{-b_{0} n_{1} ^{2} + \left ( a_{0} - b_{2} \right ) n_{1} + a_{2}}{b_{1} n_{1} - a_{1}}. \end{equation*}

**Figure 5.**
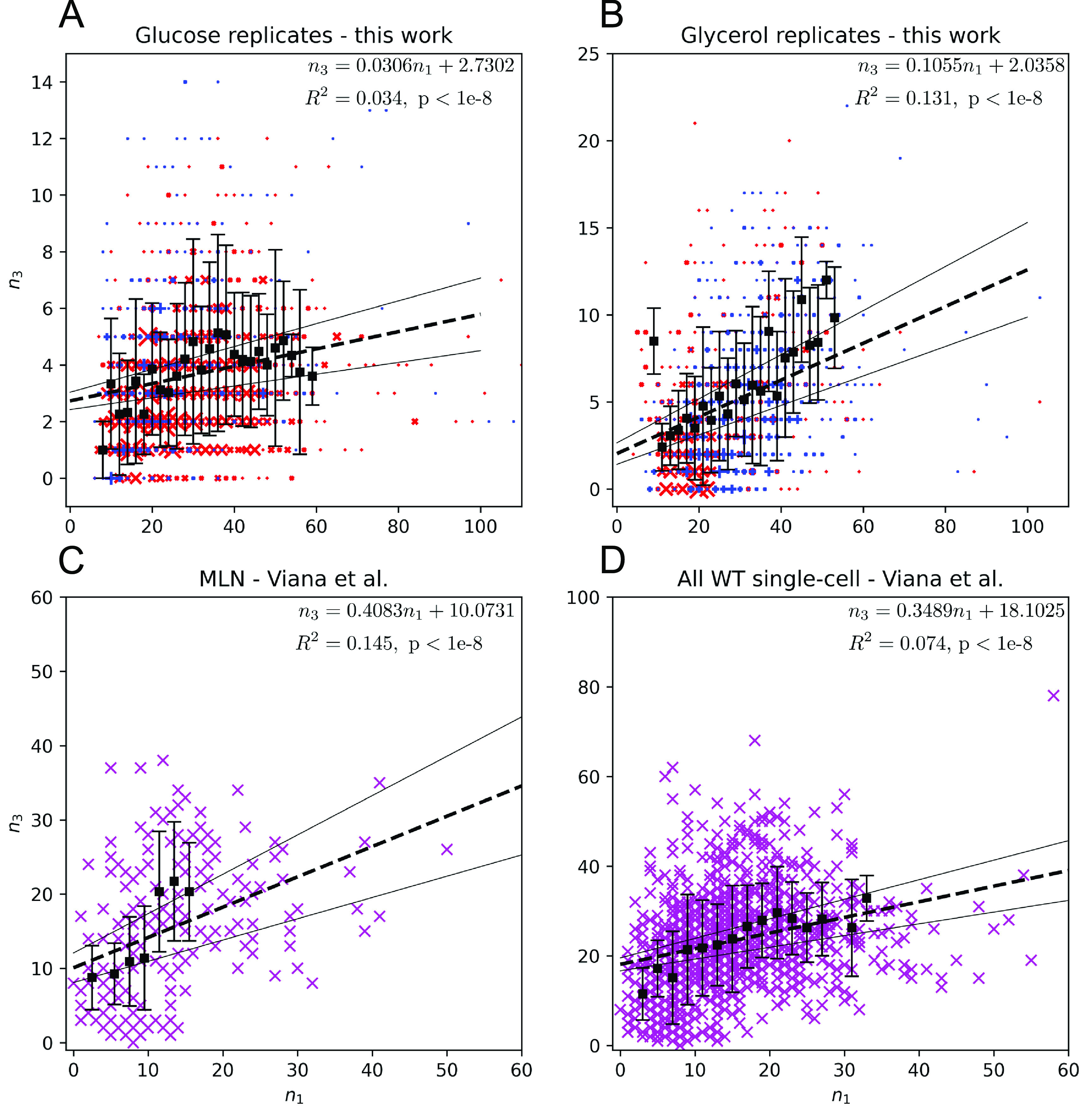
Mitochondrial networks of budding yeast (strain SRY123, mitochondria labeled with mRuby2, generous gift of S. Rafelski lab) grown in synthetic complete media with a carbon source of either glucose (A) or glycerol (B) were captured at 20 time points spaced 30 seconds apart using spinning disk confocal microscopy. Networks were converted into graphs using MitoGraph, processed using pynauty, analyzed via linear regression using statsmodels, and plotted using matplotlib and seaborn [61, 109, 123–126]. Data (magenta X’s) from a previous study [48] is shown for data subject to the mitochondria-like network (‘MLN’) definitions (section 3.1) from this work (C) as well as in its totality (D). Black boxes and error bars designate means and one standard deviation of *n*_3_ for individual values of *n*_1_. Curves of best fit are shown by thicker black dashed lines, with 95% confidence intervals in thin black lines.

Examination of *n*_3_ vs. *n*_1_ for mitochondrial networks measured in live yeast (figure 5) reveals generally positive scaling of *n*_3_ as *n*_1_ increases, implying that the leading term of a suitable model will have a positive coefficient as $n_{1} \rightarrow \infty$. The leading term of (14) implies that $n_{3} \approx \frac{-b_{0}}{b_{1}} n_{1}$ as $n_{1} \rightarrow \infty$. To satisfy the positivity requirement, either: (1) exactly one of *b*_0_ and *b*_1_ is negative and the other non-zero, or (2) at at least one of *b*_0_ and *b*_1_ is 0. If *b*_0_ is negative, fusion must be less likely as *n*_1_ increases. Alternatively, if *b*_1_ is negative, then at least one of TT- and TS-fusion must decrease in probability as both *n*_1_ and the number of edges increase. We do not believe either of these conclusions to be likely and therefore reject the model described by (14).

Therefore, we must consider the cases in which at least one of *b*_0_ and *b*_1_ is 0, and in doing so obtain the following expressions:

\begin{align*} n_{3} &amp;= \frac{b_{0}}{a_{1}} n_{1}^{2} + \frac{b_{2} - a_{0}}{a_{1}} n_{1} - \frac{a_{2}}{a_{1}} \qquad \quad &amp;b_{0} \neq b_{1}=0 \end{align*}

\begin{align*} n_{3} &amp;= -\frac{a_{0} - b_{2}}{a_{1}} n_{1} - \frac{a_{2}}{a_{1}} \qquad \quad &amp;b_{0}=b_{1}=0. \end{align*}

We note that, as any product of *n*_1_ and $E = n_{1} + 3 n_{3}$ must contain nonzero $n_{1}^{2}$ and $n_{1} n_{3}$ terms, $b_{0} = 0$ implies $b_{1} = 0$.

Following a similar approach as before for model (15), we find that $a_{1} \gt 0$, implying that fission and possibly mitophagy increase in probability in first-order as *E* increases. Conversely, in model (16) we must have either $a_{1} \lt 0$ or $a_{0} \lt b_{2}$. Both of these scenarios are permissible, so both models are potentially consistent with the data. However, fitting the data to a quadratic model with its associated constraint failed to converge, with the quadratic coefficient tending towards zero. We therefore reject the quadratic model (15) and conclude that, in this study, $b_{1} = 0$, indicating that a mass-action approach to modeling the data does not describe a mechanism of fusion that is second-order in *n*_1_ and *n*_3_. Biologically, this implies (through the lens of a mass-action framework) that TT-fusion should occur broadly while TS-fusion is either rare, slow, or (more likely) a predominantly local process. If TS-fusion is either rare or slow, outgrowth should be the primary generator of three-way junctions.

In contrast, a linear model fits each of the evaluated datasets with high significance but low *R* ^2^, implying that this model can explain only a small proportion of the data. Indeed, this model fails to capture the relatively high mean value of *n*_3_ at the lowest value of *n*_1_ as shown in figure 5(B). However, based on our assumptions, the linear model can be used to describe fission and mitophagy. This may be because fission and mitophagy are carried out using components that are not embedded in the mitochondrial membrane, potentially satisfying the well-mixed assumptions of mass-action kinetics, unlike a local fusion process. However,

We leave to a further study the exact determination and interpretation of the coefficients $a_{i},b_{j}$. However, using this approach, it may be possible to obtain values for *k*_fis_ and *k*_fis_ from the values of *a*_*i*_ and *b*_*j*_ based on network structures alone. If so, the results could be compared to directly measured experimental values of those rate constants obtained with microscopy.

### 3.3. Integration of physical properties into mitochondrial network representations

Representing mitochondria as graphs allows the powerful apparatus of graph theory to be brought to bear on this organelle, but doing so comes at the price of ignoring some aspects of shape that are likely important for real mitochondria. Two key features ignored by the graph representation are the physical lengths of the edges and the embedding of the graph in 3D space. Integrating tubule lengths or volumes into a mitochondrial network state space is likely quite useful, as doing so should introduce a conservation law on total mitochondrial volume within a cell on short timescales (i.e. before a mitophagy event) [47]. However, because length and volume are continuously varying quantities (as opposed to the discrete number of nodes and edges), state spaces integrating these quantities should also be continuously varying. Sukhorukov *et al* address this through the introduction of a minimal mitochondrial unit, which has a length of approximately $0.5~\mu\mathrm{m}$ (corresponding to the diameter at which mitochondria convert from tubules to spheroids) [68]. While this approach enables effective analysis and predictions, it also restricts mitochondrial fusion to a discrete set of positions along tubules. What other approaches could be used to combine tubule lengths and network structure into a single, cohesive framework?

One appealing candidate, persistent homology, comes from the field of applied topology. Persistent homology describes the persistence of topological features of a network, such as the number of connected components or cycles, as the scale of resolution of the representation changes [78, 127, 128]. The key idea is that ‘noisy’ features will rapidly emerge and disappear as the length scale changes, while fundamental topological features of the data should persist for much longer. To compute persistent homology, both a topological representation of the network and an associated means of describing distances within the network are selected, then a filtration parameter is varied across the range of possible distances. The output of this analysis is a set of barcodes describing the distances scales over which *n*-dimensional holes (a hole in two dimensions, a void in three dimensions, etc) in the graph are present; these barcodes can be converted into a wide range of representations with well-established associated distance metrics for the study of unweighted, weighted, and directed networks [78]. We propose here the use of persistence diagrams to summarize mitochondrial network structures at a moderate level of complexity (ignoring spatial positioning but retaining length information) and then constructing a minimal state space that captures variation within and across cells over time.

**Figure 6.**
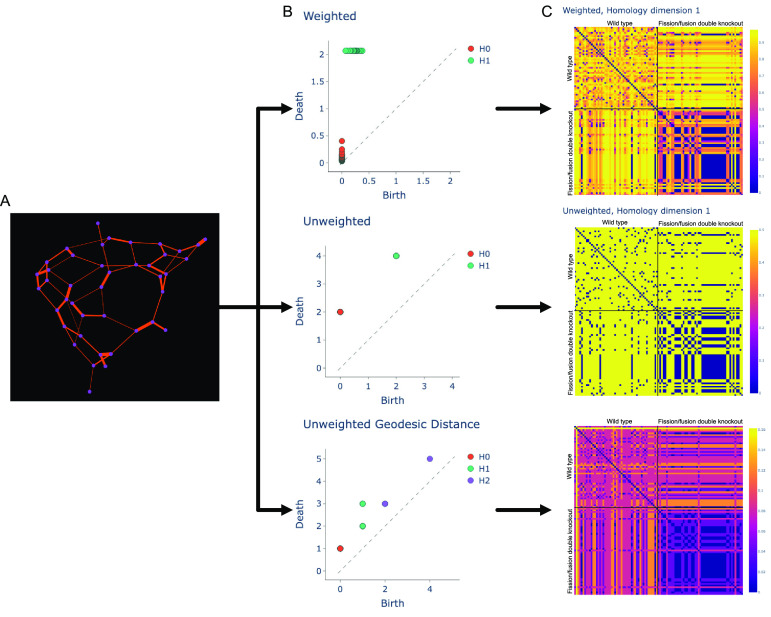
Processing the graphical structure of mitochondrial networks through a persistent homology pipeline reveals differences between the mitochondrial networks of wild-type and fission-fusion double knockout yeast. A graph extracted from a mitochondrial network (A) can be processed into PDs using measured tubule lengths (B, top, ‘weighted’) or assigning each edge a unit length (B, middle and bottom). If length is ignored, the graph can be considered either in its native unweighted form (B, middle) or converted into a fully-connected weighted graph using the graph geodesic distance (B, bottom), whose value is the shortest path between a specified pair of nodes. Single points on a persistence diagram may have multiplicity greater than 1. Once all graphs are processed, pairwise distances between PDs of different mitochondrial networks can be computed via the Bottleneck metric, leading to the generation of heatmaps (C). Data in (C) are separated into wild-type (top half of each heatmap) and double-knockout (bottom half), showing increased similarity of pairwise distances within a single strain as compared to different strains. *H*_0_, *H*_1_, and *H*_2_ refer to homology groups in 0, 1, and 2 dimensions, respectively.

We perform persistent homology analysis on mitochondrial networks as follows. As input, we take in either the unweighted or weighted graph representations of individual mitochondrial networks (figure 6(A)). From these graphs, we construct Vietoris–Rips complexes [127, 129]. Considering each node of the weighted graph as the center of a sphere, we take a parameter *ε* = 0 as the radius of the spheres and temporarily remove all of the edges from the graph. Next, we increase *ε* and reintroduce an edge to the graph once the value of *ε* reaches half the weight of that edge, i.e. when the circles of radius *ε* centered at each of the bounding nodes become tangent. Each time an edge is re-introduced, we compute the size of the homology groups of the modified graph structure. The process is equivalent for unweighted graphs, though methods for choosing how and when to reintroduce edges vary from all at once to with a delay based on some feature(s) of the nodes and edges (e.g. the presence of cycles, cliques, etc). The size of the 0th dimensional homology group counts the number of connected components of the graph. The size of the 1st homology group counts the number of cycles with at least four edges, because a three-edge loop forms the boundary of a 2-dimensional triangular simplex, which is considered to be a fundamental component for constructing simplicial complexes, rather than a void or hole. Similarly, higher dimensional homology groups have sizes corresponding to the number of higher-dimensional holes that are present [128, 130].

With this in mind, we can consider the relationship *ε* and the size of the homology group. When *ε* reaches a value at which a new member of a homology group is introduced (e.g. a 4-cycle is formed), that construct is considered to have a ‘birth’ value equal to *ϵ*. As *ε* increases further, previously generated simplices may disappear, such as when two connected components re-join into a single component, or the splitting of a cycle into two via the re-introduction of an edge. The value of *ϵ* at which a component disappears occurs is known as the ‘death’ value for that component. Once *ε* reaches the maximum edge weight of the graph, the final edge is re-introduced, and the graphical structure no longer changes. By plotting the ‘birth’ and ‘death’ values for simplices across multiple homological dimensions, known as a persistence diagram (PD), we can get a sense of how the structure of the mitochondrial network varies over its associated length scales (figure 6(B)).

It is worth noting that this analysis is not necessarily very informative for an isolated unweighted graph, as all edges are re-introduced at a single step. However, by computing the shortest path between all pairs of nodes, then constructing a graph with the original nodes and new edges between all pairs of nodes with weights corresponding to the shortest path lengths (which may be infinite), we can perform a much more informative persistent homology analysis using this shortest-path graph (figure 6(B), bottom). Once the persistence diagram is obtained, it can be used to compare two graphs and quantify their difference. Here, we use the ‘bottleneck’ distance, which is the shortest euclidean distance *d* required for a perfect matching between points on two different diagrams such that the maximum distance between any matched pair of points is *d* [131]. This method of analysis can distinguish between wild-type mitochondrial networks and those in fission/fusion double knockout yeast strains, and also suggests that morphological differences within the analyzed populations are much smaller for double knockouts than for wild-type yeast (figure 6(C)).

Although the proposed state space is approximate, it captures network features across multiple scales and may provide insight into the full, latent mitochondrial state space. Persistent homology can also be extended to study the evolution of networks over time, enabling the detection of node movement along a tubule without changes to the underlying network structure [129, 132]. Although persistent homology is not commonly used in the analysis of biological datasets, there are examples of its success in the neuroscience and protein structure literatures, as well as introductions to its practical usage and implementations in multiple programming languages.

In deciding what mathematical features to use to describe graphs, it may be important to consider what aspects of structure are biologically important so as to be sure to preserve them in the representation. Although we have chosen one way to define the placement of nodes and edges in the construction of a mitochondria-like graph, other representations (such as placing nodes at the locations of mitochondrial nucleoids and edges between nucleoids in the same connected component) may prove fruitful. Also, the embedding of a graph in three dimensional space may be extremely important in terms of how the mitochondria are able to distribute biochemical products through the cell, etc. Topology provides additional tools for comparing networks embedded into surfaces. The graph-theoretic notion of isomorphism provides a necessary but insufficient condition for evaluating the equivalence of two embedded mitochondrial network structures, as a connected component may be contained with a face of a larger and different component, which would be considered identical under isomorphism to a scenario in which the smaller connected component is on the opposite side of a cell from the larger component.

To remedy this, we can use the topological concept of isotopy with fixed vertices, which (loosely) is a continuous deformation of one graph to another that preserves the relative connectivity of the edges and vertices while not introducing new loops or edge crossings [133]. Determining whether two embedded graphs are isotopic can likely be accomplished with a linear-time algorithm based on established work [108, 134–136]. Related and likely informative approaches also include homotopy groups, persistent homotopy, and analysis of polynomial invariants of mitochondrial-like networks [137–143]. Beyond topology and persistent homology, recent advances in the study of spanning trees could be applied to any graph representation of mitochondrial networks, particularly in the study of structural differences beyond isomorphism and isotopy, as well as for the deeper study of local structural features across networks [144–148].

## 4. Biological questions raised by the study of mitochondrial networks

It is hopefully clear at this point that mitochondrial networks are interesting and fruitful targets of study for mathematicians and physicists. Why should biologists care about mitochondrial networks through the lens of mathematics?

We believe that such an approach has already proven its value, through the study of the fission-fusion double knockout in *S. cerevisiae* (Δdnm1Δfzo1). Earlier studies of this mutant demonstrated fitness defects but did not show differences in mitochondrial network structure; indeed, networks in such mutants were often referred to as indistinguishable from wild-type yeast [149, 150]. However, in recently published work, it was shown that mitochondrial networks in the double knockout strain had both altered structure and correspondingly different graph-theoretic properties as compared to the wild-type strain [48]. This is supported by our persistent homology comparisons in figure 6, which suggest that mitochondrial networks in double knockout yeast are not only distinct from wild-type but also potentially show less diversity of structures. Beyond providing a way to identify discriminate between cells with similar appearances and different fitness, graph theory also raises biological questions about the mechanism by which features of the graphs are created. These questions thus require a combined biological and mathematical approach.

For example, as the experimental biology toolkit has grown, it has become increasingly clear that mitochondrial networks serve as integrators of cellular information, changing dynamics and structure in response to both externally and internally induced changes in cellular state [40, 41]. How is the information integrated and then translated into changes in mitochondrial morphology? Which small molecules, proteins, post-translational modifications, and pathways are involved, and how do they communicate this information? How does inter-organelle interaction impact mitochondrial function, structure, and dynamics (and vice versa)[19, 21, 151]?

In *S. cerevisiae*, a daughter bud inherits its mitochondria from the mother cell. How do the mitochondrial network structures sampled by the bud compare to those of the mother? Extrapolating further, how do the mitochondrial networks sampled by a single cell in an isogenic population compare to those seen across the entire population? These questions are of more than just academic interest: population heterogeneity, especially of a non-genetic nature, is relevant to biological systems ranging from embryogenesis to tumor proliferation, in addition to the much more specific case of mtDNA heteroplasmy (which is itself associated with detrimental health conditions) [152, 153]. Dissecting the basis of cellular heterogeneity can be done via comparison of trajectories in state space, through evaluation of both state sampling probability (how often do we find cells in state *X*?) and transition rate (how often does a cell move from state *X* to state *Y*?). Mathematics and physics provide a means of constructing cellular state spaces via a potential landscape from experimental data, from which ‘force’ fields can be constructed, and these landscapes then raise the question of what molecular pathways determine their shape. This approach has proven useful in studies ranging from stem cell differentiation (where the analogous concept of Waddington’s landscape is often discussed), to flagellar beating, to cellular motility [154–160]. We believe that the examination of mitochondrial networks over time in large populations of cells can provide insight into the general principles guiding cellular heterogeneity: data collection is non-terminal, state spaces can be defined in reasonable ways (graph theoretic or otherwise), and the features being measured are deeply and intrinsically linked to cellular state.

**Figure 7.**
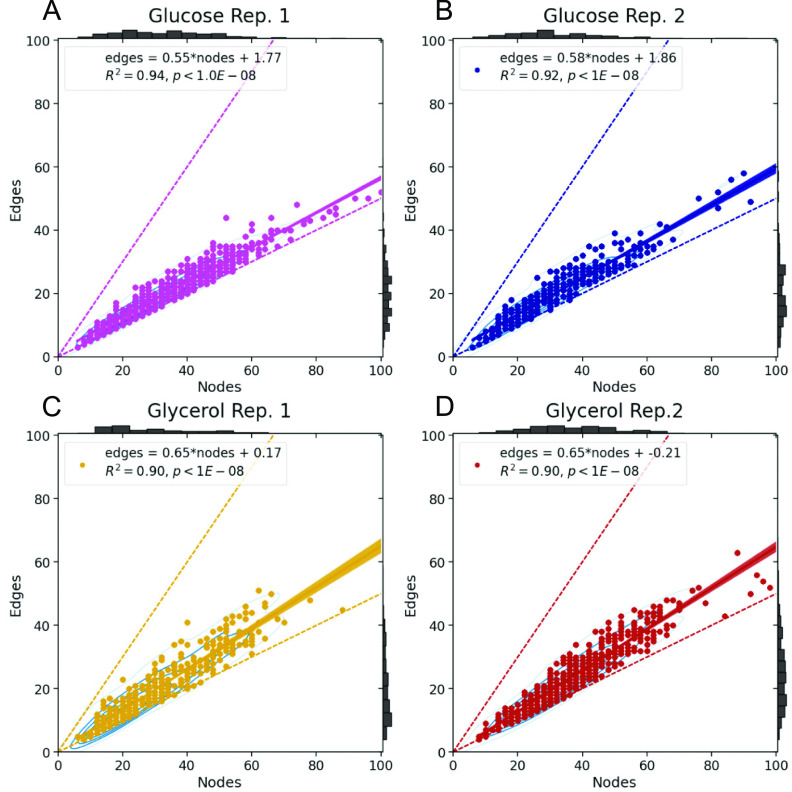
Linear scaling of edges with nodes in mitochondrial networks. The dashed lines in each panel demarcate the theoretical edge-node scaling limits of slope $\frac{1}{2}$ (lower) and $\frac{3}{2}$ (upper).

Another notable feature of mitochondrial networks is that the processes of mitochondrial fission and fusion appear reciprocally regulate one another [161]. This, too, is worthy of deeper investigation: in figure 7, we show that, for mitochondrial networks of isogenic *S. cerevisiae* grown in synthetic complete media with either glucose or glycerol, the number of edges scales linearly with the number of nodes. For the majority of the (hundreds of) data points shown in each subpanel, the regression intercepts are negligible, providing another mathematical result: the slope of the regression can be understood as half the average degree of a ‘typical’ mitochondrial network (or collection of networks) in the population, a result obtained by dividing both sides of (1) by $2|N|$. We can thereby establish boundaries for the permissible region of mitochondrial edge-node state space by setting all of the node degrees to either 1 or 3, resulting in slopes of $\frac{1}{2}$ and $\frac{3}{2}$, respectively (lower and upper dashed lines in all panels of figure 7). Were we to consider all planar graphs, combining (1) with (2) would loosen the upper bound for the slope to 3.

That the linear relation shows a constant slope for all *N* indicates the average degree of the graph is maintained as the graph grows. This finding, in combination with the reciprocal regulation referenced above, strongly hints at some sort of homeostatic mechanism for keeping some feature or features of the network (in this case, the average degree) close to some set point. One way to detect and study this mechanism is to compare the observed distribution of graphs with the distribution predicted from the morphological operations—if cells only display a small subset of the possible mitochondria-like graphs expected from the known morphological processes, this would be a clear hallmark of homeostasis. What are the molecular players and relevant pathways in this regulatory mechanism, and what function does it serve?

Finally, the approach discussed above of relating graph distributions to the rates of mitochondrial morphological processes raises the possibility that it might be the morphological processes themselves, rather than the resulting graph structures, that are biologically significant. For example, fission and fusion might have as their primary roles the partitioning of mitochondrial genomes, with the resulting graph just a byproduct of this role.

Mitochondrial networks can be considered as fundamental demonstrations of graph theory and topology in living systems. This link is neither trivial nor unidirectional: the structure of the network is heavily regulated by both mathematics and the cell. Morphological dynamics modify the structure of mitochondrial networks, but seemingly do so in a restricted way that possibly maintains some homeostatic principle that remains unknown. A greater understanding of both the mathematical characteristics of mitochondrial-like networks and the biological regulatory mechanisms that guide mitochondrial network structure evolution will provide insight into this cellular information integrator, its responses to changes in cellular state, and the relationships between network function and physiology.

## Data Availability

The data that support the findings of this study are openly available at the following URL/DOI: https://doi.org/10.7272/Q6PR7T7K and https://datadryad.org/stash/share/UYDIfFi-b0ZT1r8aFbBPbA2Tr2DmzZ60IZ6-WRhwhvA.

## References

[1] Thompson D W (1992). On Growth and Form Canto.

[2] Karnkowska A (2016). A eukaryote without a mitochondrial organelle. Curr. Biol..

[3] Zhang Z-W, Cheng J, Xu F, Chen Y-E, Du J-B, Yuan M, Zhu F, Xu X-C, Yuan S (2011). Red blood cell extrudes nucleus and mitochondria against oxidative stress. IUBMB Life.

[4] Green D R, Reed J C (1998). Mitochondria and apoptosis. Science.

[5] Wang C, Youle R J (2009). The role of mitochondria in apoptosis. Annu. Rev. Genet..

[6] De Stefani D, Rizzuto R, Pozzan T (2016). Enjoy the trip: calcium in mitochondria back and forth. Annu. Rev. Biochem..

[7] Rossi A, Pizzo P, Filadi R (2019). Calcium, mitochondria and cell metabolism: a functional triangle in bioenergetics. Biochim. Biophys. Acta.

[8] Mayr J A (2015). Lipid metabolism in mitochondrial membranes. J. Inherit. Metab. Dis..

[9] Bradley J, Swann K (2019). Mitochondria and lipid metabolism in mammalian oocytes and early embryos. Int. J. Dev. Biol..

[10] Diano S, Horvath T L (2012). Mitochondrial uncoupling protein 2 (UCP2) in glucose and lipid metabolism. Trends Mol. Med..

[11] Shadel G S, Horvath T L (2015). Mitochondrial ROS signaling in organismal homeostasis. Cell.

[12] Paillusson S, Stoica R, Gomez-Suaga P, Lau D H W, Mueller S, Miller T, Miller C C J (2016). There’s something wrong with my MAM; the ER–mitochondria axis and neurodegenerative diseases. Trends Neurosci..

[13] Rackham O, Filipovska A (2022). Organization and expression of the mammalian mitochondrial genome. Nat. Rev. Genet..

[14] West A P, Shadel G S (2017). Mitochondrial DNA in innate immune responses and inflammatory pathology. Nat. Rev. Immunol..

[15] Wu Z, Sainz A G, Shadel G S (2021). Mitochondrial DNA: cellular genotoxic stress sentinel. Trends Biochem. Sci..

[16] Howell N, Chinnery P, Ghosh S, Fahy E, Turnbull D (2000). Transmission of the human mitochondrial genome. Hum. Reprod..

[17] Shokolenko I N, Alexeyev M F (2015). Mitochondrial DNA: a disposable genome?. Biochim. Biophys. Acta.

[18] Milo R, Jorgensen P, Moran U, Weber G, Springer M (2010). BNID 107091 in bionumbers—the database of key numbers in molecular and cell biology. Nucleic Acids Res..

[19] Friedman J R, Lackner L L, West M, DiBenedetto J R, Nunnari J, Voeltz G K (2011). ER tubules mark sites of mitochondrial division. Science.

[20] Lewis S C, Uchiyama L F, Nunnari J (2016). ER-mitochondria contacts couple mtDNA synthesis with mitochondrial division in human cells. Science.

[21] Abrisch R G, Gumbin S C, Wisniewski B T, Lackner L L, Voeltz G K (2020). Fission and fusion machineries converge at ER contact sites to regulate mitochondrial morphology. J. Cell Biol..

[22] Qin J (2020). ER-mitochondria contacts promote mtDNA nucleoids active transportation via mitochondrial dynamic tubulation. Nat. Commun..

[23] Kleele T (2021). Distinct fission signatures predict mitochondrial degradation or biogenesis. Nature.

[24] Nunnari J, Marshall W F, Straight A, Murray A, Sedat J W, Walter P (1997). Mitochondrial transmission during mating in saccharomyces cerevisiae is determined by mitochondrial fusion and fission and the intramitochondrial segregation of mitochondrial DNA. Mol. Biol. Cell.

[25] Friedman J R, Nunnari J (2014). Mitochondrial form and function. Nature.

[26] Murley A, Lackner L L, Osman C, West M, Voeltz G K, Walter P, Nunnari J (2013). ER-associated mitochondrial division links the distribution of mitochondria and mitochondrial DNA in yeast. eLife.

[27] DeVay R M, Dominguez-Ramirez L, Lackner L L, Hoppins S, Stahlberg H, Nunnari J (2009). Coassembly of Mgm1 isoforms requires cardiolipin and mediates mitochondrial inner membrane fusion. J. Cell Biol..

[28] Mears J A, Lackner L L, Fang S, Ingerman E, Nunnari J, Hinshaw J E (2011). Conformational changes in Dnm1 support a contractile mechanism for mitochondrial fission. Nat. Struct. Mol. Biol..

[29] Kraft L M, Lackner L L (2017). Mitochondria-driven assembly of a cortical anchor for mitochondria and dynein. J. Cell Biol..

[30] Scorrano L (2019). Coming together to define membrane contact sites. Nat. Commun..

[31] Schmit H L, Kraft L M, Lee-Smith C F, Lackner L L (2018). The role of mitochondria in anchoring dynein to the cell cortex extends beyond clustering the anchor protein. Cell Cycle.

[32] Hoppins S, Nunnari J (2009). The molecular mechanism of mitochondrial fusion. Biochim. Biophys. Acta.

[33] Kornmann B, Currie E, Collins S R, Schuldiner M, Nunnari J, Weissman J S, Walter P (2009). An ER-mitochondria tethering complex revealed by a synthetic biology screen. Science.

[34] Naylor K, Ingerman E, Okreglak V, Marino M, Hinshaw J E, Nunnari J (2006). Mdv1 interacts with assembled Dnm1 to promote mitochondrial division. J. Biol. Chem..

[35] Bleazard W, McCaffery J M, King E J, Bale S, Mozdy A, Tieu Q, Nunnari J, Shaw J M (1999). The dynamin-related GTPase Dnm1 regulates mitochondrial fission in yeast. Nat. Cell Biol..

[36] Hermann G J, Thatcher J W, Mills J P, Hales K G, Fuller M T, Nunnari J, Shaw J M (1998). Mitochondrial fusion in yeast requires the transmembrane GTPase Fzo1p. J. Cell Biol..

[37] Ingerman E, Perkins E M, Marino M, Mears J A, McCaffery J M, Hinshaw J E, Nunnari J (2005). Dnm1 forms spirals that are structurally tailored to fit mitochondria. J. Cell Biol..

[38] Hoitzing H, Johnston I G, Jones N S (2015). What is the function of mitochondrial networks? A theoretical assessment of hypotheses and proposal for future research. BioEssays.

[39] Mitra K, Wunder C, Roysam B, Lin G, Lippincott-Schwartz J (2009). A hyperfused mitochondrial state achieved at G1–S regulates cyclin E buildup and entry into S phase. Proc. Natl Acad. Sci. USA.

[40] Picard M, Shirihai O S, Gentil B J, Burelle Y (2013). Mitochondrial morphology transitions and functions: implications for retrograde signaling?. Am. J. Physiol. Regul. Integr. Comp. Physiol..

[41] Eisner V, Picard M, Hajnóczky G (2018). Mitochondrial dynamics in adaptive and maladaptive cellular stress responses. Nat. Cell Biol..

[42] Bereiter-Hahn J, Jendrach M, Jeon K W (2010). Mitochondrial dynamics. International Review of Cell and Molecular Biology.

[43] Cosentino K, García-Sáez A J (2014). Mitochondrial alterations in apoptosis. Chem. Phys. Lipids.

[44] Banerjee R, Joshi N, Nagotu S (2020). Cell organelles and yeast longevity: an intertwined regulation. Curr. Genet..

[45] Brown A I, Westrate L M, Koslover E F (2020). Impact of global structure on diffusive exploration of organelle networks. Sci. Rep..

[46] Scott Z C, Brown A I, Mogre S S, Westrate L M, Koslover E F (2021). Diffusive search and trajectories on tubular networks: a propagator approach. Eur. Phys. J. E.

[47] Rafelski S M, Viana M P, Zhang Y, Chan Y H M, Thorn K S, Yam P, Fung J C, Li H, Costa L d F, Marshall W F (2012). Mitochondrial network size scaling in budding yeast. Science.

[48] Viana M P, Brown A I, Mueller I A, Goul C, Koslover E F, Rafelski S M (2020). Mitochondrial fission and fusion dynamics generate efficient, robust and evenly distributed network topologies in budding yeast cells. Cell Syst..

[49] Zamponi N, Zamponi E, Cannas S A, Billoni O V, Helguera P R, Chialvo D R (2018). Mitochondrial network complexity emerges from fission/fusion dynamics. Sci. Rep..

[50] Zamponi N, Zamponi E, Cannas S A, Chialvo D R (2022). Universal dynamics of mitochondrial networks: a finite-size scaling analysis. Sci. Rep..

[51] Botstein D, Fink G R (2011). Yeast: an experimental organism for 21st century biology. Genetics.

[52] Stovicek V, Holkenbrink C, Borodina I (2017). CRISPR/Cas system for yeast genome engineering: advances and applications. FEMS Yeast Res..

[53] Lian J, Mishra S, Zhao H (2018). Recent advances in metabolic engineering of saccharomyces cerevisiae: new tools and their applications. Metab. Eng..

[54] Karathia H, Vilaprinyo E, Sorribas A, Alves R (2011). Saccharomyces cerevisiae as a model organism: a comparative study. PLoS One.

[55] Giaever G, Nislow C (2014). The yeast deletion collection: a decade of functional genomics. Genetics.

[56] Hinnebusch A G, Johnston M (2011). YeastBook: an encyclopedia of the reference eukaryotic cell. Genetics.

[57] Duina A A, Miller M E, Keeney J B (2014). Budding yeast for budding geneticists: a primer on the saccharomyces cerevisiae model system. Genetics.

[58] Ceccatelli Berti C, di Punzio G, Dallabona C, Baruffini E, Goffrini P, Lodi T, Donnini C (2021). The power of yeast in modelling human nuclear mutations associated with mitochondrial diseases. Genes.

[59] Merz S, Westermann B (2009). Genome-wide deletion mutant analysis reveals genes required for respiratory growth, mitochondrial genome maintenance and mitochondrial protein synthesis in saccharomyces cerevisiae. Genome Biol..

[60] Gilea A I, Ceccatelli Berti C, Magistrati M, di Punzio G, Goffrini P, Baruffini E, Dallabona C (2021). Saccharomyces cerevisiae as a tool for studying mutations in nuclear genes involved in diseases caused by mitochondrial DNA instability. Genes.

[61] Viana M P, Lim S, Rafelski S M, Paluch E K (2015). Quantifying mitochondrial content in living cells. Methods in Cell Biology (Biophysical Methods in Cell Biology vol 125).

[62] Harwig M C, Viana M P, Egner J M, Harwig J J, Widlansky M E, Rafelski S M, Hill R B (2018). Methods for imaging mammalian mitochondrial morphology: a prospective on MitoGraph. Anal. Biochem..

[63] Jakobs S, Stephan T, Ilgen P, Brüser C (2020). Light microscopy of mitochondria at the nanoscale. Annu. Rev. Biophys..

[64] Bereiter-Hahn J, Vöth M (1994). Dynamics of mitochondria in living cells: shape changes, dislocations, fusion and fission of mitochondria. Microsc. Res. Tech..

[65] Kuznetsov A V, Hermann M, Saks V, Hengster P, Margreiter R (2009). The cell-type specificity of mitochondrial dynamics. Int. J. Biochem. Cell Biol..

[66] Rohani A, Kashatus J A, Sessions D T, Sharmin S, Kashatus D F (2020). Mito hacker: a set of tools to enable high-throughput analysis of mitochondrial network morphology. Sci. Rep..

[67] Chacko L A, Ananthanarayanan V (2019). Quantification of mitochondrial dynamics in fission yeast. Bio Protoc..

[68] Sukhorukov V M, Dikov D, Reichert A S, Meyer-Hermann M (2012). Emergence of the mitochondrial reticulum from fission and fusion dynamics. PLoS Comput. Biol..

[69] Aon M A, Cortassa S, O’Rourke B (2004). Percolation and criticality in a mitochondrial network. Proc. Natl Acad. Sci. USA.

[70] Rivier N (1990). Geometry and fluctuations of surfaces. J. Phys. Colloq..

[71] West D B (2018). Introduction to Graph Theory (Pearson Modern Classic).

[72] Adhikari M R (2016). Basic Algebraic Topology and its Applications.

[73] Altmann R (1894). Die Elementarorganismen und Ihre Beziehungen zu den Zellen.

[74] Ernster L, Schatz G (1981). Mitochondria: a historical review. J. Cell Biol..

[75] Meyer C D (2000). Matrix Analysis and Applied Linear Algebra.

[76] Frigg R, Berkovitz J, Kronz F (2020). The ergodic hierarchy. https://plato.stanford.edu/archives/fall2020/entries/ergodic-hierarchy/.

[77] Munkres J R (2000). Topology.

[78] Ghrist R (2007). Barcodes: the persistent topology of data. Bull. New Ser. Am. Math. Soc..

[79] Grbic J, Wu J, Xia K, Wei G (2022). Aspects of topological approaches for data science. Found. Data Sci..

[80] Chen B-C (2014). Lattice light-sheet microscopy: imaging molecules to embryos at high spatiotemporal resolution. Science.

[81] Guo Y (2018). Visualizing intracellular organelle and cytoskeletal interactions at nanoscale resolution on millisecond timescales. Cell.

[82] Manley S, Gillette J M, Patterson G H, Shroff H, Hess H F, Betzig E, Lippincott-Schwartz J (2008). High-density mapping of single-molecule trajectories with photoactivated localization microscopy. Nat. Methods.

[83] York A G, Chandris P, Nogare D D, Head J, Wawrzusin P, Fischer R S, Chitnis A, Shroff H (2013). Instant super-resolution imaging in live cells and embryos via analog image processing. Nat. Methods.

[84] Huang X (2018). Fast, long-term, super-resolution imaging with Hessian structured illumination microscopy. Nat. Biotechnol..

[85] Prakash K, Diederich B, Heintzmann R, Schermelleh L (2022). Super-resolution microscopy: a brief history and new avenues. Phil. Trans. R. Soc. A.

[86] Ouellet M, Guillebaud G, Gervais V, St-Pierre D L, Germain M (2017). A novel algorithm identifies stress-induced alterations in mitochondrial connectivity and inner membrane structure from confocal images. PLoS Comput. Biol..

[87] Kandel J, Chou P, Eckmann D M (2015). Automated detection of whole-cell mitochondrial motility and its dependence on cytoarchitectural integrity. Biotechnol. Bioeng..

[88] Lihavainen E, Mäkelä J, Spelbrink J N, Ribeiro A S (2012). Mytoe: automatic analysis of mitochondrial dynamics. Bioinformatics.

[89] Fischer C A, Besora-Casals L, Rolland S G, Haeussler S, Singh K, Duchen M, Conradt B, Marr C (2020). MitoSegNet: easy-to-use deep learning segmentation for analyzing mitochondrial morphology. iScience.

[90] Valente A J, Maddalena L A, Robb E L, Moradi F, Stuart J A (2017). A simple ImageJ macro tool for analyzing mitochondrial network morphology in mammalian cell culture. Acta Histochem..

[91] Lefebvre A E Y T, Ma D, Kessenbrock K, Lawson D A, Digman M A (2021). Automated segmentation and tracking of mitochondria in live-cell time-lapse images. Nat. Methods.

[92] Barthélemy M (2011). Spatial networks. Phys. Rep..

[93] Holme P, Saramäki J (2012). Temporal networks. Phys. Rep..

[94] Kivelä M, Arenas A, Barthelemy M, Gleeson J P, Moreno Y, Porter M A (2014). Multilayer networks. J. Complex Netw..

[95] Sukhorukov V M, Meyer-Hermann M (2015). Structural heterogeneity of mitochondria induced by the microtubule cytoskeleton. Sci. Rep..

[96] Mouli P K, Twig G, Shirihai O S (2009). Frequency and Selectivity of mitochondrial fusion are key to its quality maintenance function. Biophys. J..

[97] Patel P K, Shirihai O, Huang K C (2013). Optimal dynamics for quality control in spatially distributed mitochondrial networks. PLoS Comput. Biol..

[98] Hoitzing H, Johnston I G, Jones N S, Holcman D (2017). Stochastic models for evolving cellular populations of mitochondria: disease, development and ageing. Stochastic Processes, Multiscale Modeling and Numerical Methods for Computational Cellular Biology.

[99] Aryaman J, Bowles C, Jones N S, Johnston I G (2019). Mitochondrial network state scales mtDNA genetic dynamics. Genetics.

[100] Chustecki J M, Gibbs D J, Bassel G W, Johnston I G (2021). Network analysis of arabidopsis mitochondrial dynamics reveals a resolved tradeoff between physical distribution and social connectivity. Cell Syst..

[101] Glastad R C, Johnston I G (2023). Mitochondrial network structure controls cell-to-cell mtDNA variability generated by cell divisions. PLoS Comput. Biol..

[102] Hill M, Agarwal M, Calloway C, Niederhut D, Shupe D (2020). Spectral analysis of mitochondrial dynamics: a graph-theoretic approach to understanding subcellular pathology.

[103] Hill M D (2022). Modeling and analysis of mitochondrial dynamics using dynamic social network graphs. PhD Thesis.

[104] Fazli M, Hill M, Durden A, Mattson R, Loy A T, Reaves B, Courtney A, Quinn F D, Chennubhotla C, Quinn S (2020). OrNet—a Python toolkit to model the diffuse structure of organelles as social networks. J. Open Source Softw..

[105] Pulagam N, Hill M, Fazli M, Mattson R, Zain M, Durden A, Quinn F D, Chennubhotla S C, Quinn S P (2021). Classification of diffuse subcellular morphologies.

[106] Jasiński J (2013). Ramsey degrees of boron tree structures. Combinatorica.

[107] Babai L (2016). Graph isomorphism in quasipolynomial time. https://arxiv.org/abs/1512.03547.

[108] Hopcroft J E, Wong J K (1974). Linear time algorithm for isomorphism of planar graphs (preliminary report).

[109] McKay B D, Piperno A (2014). Practical graph isomorphism, II. J. Sym. Comp..

[110] Bonichon N, Gavoille C, Hanusse N, Poulalhon D, Schaeffer G (2006). Planar graphs, via well-orderly maps and trees. Graphs Comb..

[111] Bodirsky M, Fusy E, Kang M, Vigerske S (2006). Enumeration of unlabeled outerplanar graphs. https://arxiv.org/abs/math/0511422.

[112] Sloane N J A (2022). Entry A005964 in the on-line encyclopedia of integer sequences. https://oeis.org/A005964.

[113] Sloane N J A (2022). Entry A000672 in the on-line encyclopedia of integer sequences. https://oeis.org/A005964.

[114] Lauber J K (1982). Retinal pigment epithelium: ring mitochondria and lesions induced by continuous light. Curr. Eye Res..

[115] Funk R, Nagel F, Wonka F, Krinke H, Gölfert F, Hofer A (1999). Effects of heat shock on the functional morphology of cell organelles observed by video-enhanced microscopy. Anat. Rec..

[116] Liu X, Hajnóczky G (2011). Altered fusion dynamics underlie unique morphological changes in mitochondria during hypoxia–reoxygenation stress. Cell Death Differ..

[117] Guo S, Ma Y, Pan Y, Smith Z J, Chu K (2021). Organelle-specific phase contrast microscopy enables gentle monitoring and analysis of mitochondrial network dynamics. Biomed. Opt. Express.

[118] Lambiotte R, Rosvall M, Scholtes I (2019). From networks to optimal higher-order models of complex systems. Nat. Phys..

[119] Ghoshal G, Zlatić V, Caldarelli G, Newman M E J (2009). Random hypergraphs and their applications. Phys. Rev. E.

[120] Berge C (1989). Hypergraphs: Combinatorics of Finite Sets (North-Holland Mathematical Library).

[121] Zwillinger D (2002). CRC Standard Mathematical Tables and Formulae.

[122] Ouvrard X (2020). Hypergraphs: an introduction and review. https://arxiv.org/abs/2002.05014.

[123] Dobsan P (2021). Pynauty original-date: 28 January 2021 T22:37:59Z. https://github.com/pdobsan/pynauty.

[124] Seabold S, Perktold J (2010). Statsmodels: econometric and statistical modeling with Python.

[125] Hunter J D (2007). Matplotlib: a 2D graphics environment. Comput. Sci. Eng..

[126] Waskom M L (2021). seaborn: statistical data visualization. J. Open Source Softw..

[127] Bubenik P (2015). Statistical topological data analysis using persistence landscapes. J. Mach. Learn. Res..

[128] Edelsbrunner H, Harer J L (2022). Computational Topology: An Introduction.

[129] Hajij M, Wang B, Scheidegger C, Rosen P (2017). Persistent homology guided exploration of time-varying graphs. https://arxiv.org/abs/1707.06683.

[130] Horak D, Maletić S, Rajković M (2009). Persistent homology of complex networks. J. Stat. Mech..

[131] Kerber M, Morozov D, Nigmetov A (2017). Geometry helps to compare persistence diagrams. ACM J. Exp. Algorithmics.

[132] Ravishanker N, Chen R (2021). An introduction to persistent homology for time series. Wiley Interdiscip. Rev. Comput. Stat..

[133] de Verdière E C (2017). Computational topology of graphs on surfaces. Handbook of Discrete and Computational Geometry.

[134] de Verdière E C, de Mesmay A (2014). Testing graph isotopy on surfaces. Discrete Comput. Geom..

[135] Hopcroft J E, Tarjan R E, Miller R E, Thatcher J W, Bohlinger J D (1972). Isomorphism of planar graphs (working paper).

[136] de Mesmay A (2018). Answer to “complexity of isotopy of embedded graphs”. https://cstheory.stackexchange.com/a/41552.

[137] Babson E, Barcelo H, de Longueville M, Laubenbacher R (2006). Homotopy theory of graphs. J. Algebr. Comb..

[138] Chen B, Yau S-T, Yeh Y-N (2001). Graph homotopy and Graham homotopy. Discrete Math..

[139] Haarmann J, Murphy M P, Peters C S, Staecker P C (2015). Homotopy equivalence in finite digital images. J. Math. Imaging Vis..

[140] Noy M (2003). Graphs determined by polynomial invariants. Theor. Comput. Sci..

[141] Evako A V (2015). Classification of digital n-manifolds. Discrete Appl. Math..

[142] Negami S (1987). Polynomial invariants of graphs. Trans. Am. Math. Soc..

[143] Kauffman L H (1989). Invariants of graphs in three-space. Trans. Am. Math. Soc..

[144] Bazlamaçcı C F, Hindi K S (2001). Minimum-weight spanning tree algorithms a survey and empirical study. Comput. Oper. Res..

[145] Evako A V (2017). Classification of graphs based on homotopy equivalence. Homotopy equivalent graphs. Basic graphs and complexity of homotopy equivalence classes of graphs. https://arxiv.org/abs/1512.07989.

[146] Gargano L, Hell P, Stacho L, Vaccaro U, Widmayer P, Eidenbenz S, Triguero F, Morales R, Conejo R, Hennessy M (2002). Spanning trees with bounded number of branch vertices. Automata, Languages and Programming (Lecture Notes in Computer Science).

[147] Zhang R, Kabadi S N, Punnen A P (2011). The minimum spanning tree problem with conflict constraints and its variations. Discrete Optim..

[148] Ozen M, Lesaja G, Wang H (2020). Globally optimal dense and sparse spanning trees and their applications. Stat. Optim. Inf. Comput..

[149] Sesaki H, Southard S M, Yaffe M P, Jensen R E (2003). Mgm1p, a dynamin-related GTPase, is essential for fusion of the mitochondrial outer membrane. Mol. Biol. Cell.

[150] Bernhardt D, Müller M, Reichert A S, Osiewacz H D (2015). Simultaneous impairment of mitochondrial fission and fusion reduces mitophagy and shortens replicative lifespan. Sci. Rep..

[151] Westrate L M, Drocco J A, Martin K R, Hlavacek W S, MacKeigan J P (2014). Mitochondrial morphological features are associated with fission and fusion events. PLoS One.

[152] Huang S (2009). Non-genetic heterogeneity of cells in development: more than just noise. Development.

[153] Aryaman J, Johnston I G, Jones N S (2019). Mitochondrial heterogeneity. Front. Genet..

[154] Chang A Y, Marshall W F (2019). Dynamics of living cells in a cytomorphological state space. Proc. Natl Acad. Sci. USA.

[155] Wang P, Song C, Zhang H, Wu Z, Tian X-J, Xing J (2014). Epigenetic state network approach for describing cell phenotypic transitions. Interface Focus.

[156] Waddington C H (1957). The Strategy of the Genes: A Discussion of Some Aspects of Theoretical Biology.

[157] Qiu X (2022). Mapping transcriptomic vector fields of single cells. Cell.

[158] Wang W, Poe D, Yang Y, Hyatt T, Xing J (2022). Epithelial-to-mesenchymal transition proceeds through directional destabilization of multidimensional attractor. eLife.

[159] Larson B T, Garbus J, Pollack J B, Marshall W F (2022). A unicellular walker controlled by a microtubule-based finite-state machine. Curr. Biol..

[160] Kimmel J C, Chang A Y, Brack A S, Marshall W F (2018). Inferring cell state by quantitative motility analysis reveals a dynamic state system and broken detailed balance. PLoS Comput. Biol..

[161] Sabouny R, Shutt T E (2020). Reciprocal regulation of mitochondrial fission and fusion. Trends Biochem. Sci..

